# Antimicrobial stewardship in the UK during the COVID-19 pandemic: a population-based cohort study and interrupted time-series analysis

**DOI:** 10.3399/BJGP.2020.1051

**Published:** 2021-04-07

**Authors:** Emma Rezel-Potts, Veline L’Esperance, Martin C Gulliford

**Affiliations:** School of Population Health and Environmental Sciences, King’s College London, London; National Institute for Health Research Biomedical Research Centre (BRC), Guy’s and St Thomas’ NHS Foundation Trust, London.; School of Population Health and Environmental Sciences, King’s College London, London.; School of Population Health and Environmental Sciences, King’s College London; National Institute for Health Research (NIHR) Biomedical Research Centre (BRC), Guy’s and St Thomas’ NHS Foundation Trust, London.

**Keywords:** antibiotic prescribing, antimicrobial stewardship, COVID-19, respiratory tract infections, SARS-CoV-2, urinary tract infections

## Abstract

**Background:**

The COVID-19 pandemic has altered the context for antimicrobial stewardship in primary care.

**Aim:**

To assess the effect of the pandemic on antibiotic prescribing, accounting for changes in consultations for respiratory and urinary tract infections (RTIs/UTIs).

**Design and setting:**

Population-based cohort study using the UK Clinical Practice Research Datalink (CPRD) GOLD database from January 2017 to September 2020.

**Method:**

Interrupted time-series analysis evaluated changes in antibiotic prescribing and RTI/UTI consultations adjusting for age, sex, season, and secular trends. The authors assessed the proportion of COVID-19 episodes associated with antibiotic prescribing.

**Results:**

There were 253 655 registered patients in 2017 and 232 218 in 2020, with 559 461 antibiotic prescriptions, 216 110 RTI consultations, and 36 402 UTI consultations. Compared with prepandemic months, March 2020 was associated with higher antibiotic prescribing (adjusted rate ratio [ARR] 1.13; 95% confidence interval [CI] = 1.11 to 1.16). Antibiotic prescribing fell below predicted rates between April and August 2020, reaching a minimum in May (ARR 0.73; 95% CI = 0.71 to 0.75). Pandemic months were associated with lower rates of RTI/UTI consultations, particularly in April for RTIs (ARR 0.23; 95% CI = 0.22 to 0.25). There were small reductions in the proportion of RTI consultations with antibiotic prescribed and no reduction for UTIs. Among 25 889 COVID-19 patients, 2942 (11%) had antibiotics within a COVID-19 episode.

**Conclusion:**

Pandemic months were initially associated with increased antibiotic prescribing, which then fell below expected levels during the national lockdown. Findings are reassuring that antibiotic stewardship priorities have not been neglected because of COVID-19. Research is required into the effects of reduced RTI/UTI consultations on incidence of serious bacterial infections.

## INTRODUCTION

Antibiotic prescribing in primary care accounts for 70% of medical antibiotic use,^1^ and is considered to be a significant driver of antimicrobial resistance.^2^^,^^3^ Over the last decade a concerted and multifaceted effort has been made to reduce unnecessary and inappropriate antibiotic prescribing in primary care.^2^^,^^4^ This has contributed to a 13.7% reduction in antibiotic prescriptions from 2015 to 2019.

In primary care, respiratory tract infections (RTIs) and urinary tract infections (UTIs) are the most frequent indications for antibiotic prescription.^5^^–^^7^ Analysis of trends in antibiotic prescribing rates from 2002 to 2017 found that reductions began earlier and were greatest in magnitude for RTIs, while there have been comparatively modest and recent reductions for other indications.^7^ UTIs are more frequently associated with complications,^8^^–^^10^ and appropriate antibiotic selection, rather than antibiotic reduction, may be a greater focus for these conditions.^1^

During 2020, the COVID-19 pandemic associated with the SARS-CoV-2 virus profoundly altered the context for antimicrobial stewardship in primary care. The SARS-CoV-2 virus causes illness that can affect multiple organ systems. However, the initial presentation is usually with respiratory symptoms, including fever and cough, which may progress to pneumonia and respiratory failure in severe cases. Antibiotic therapy for respiratory infections in primary care is typically empirical, guided by clinical judgement rather than microbiological findings, and is commonly prescribed for community-acquired cases of pneumonia and ‘chest infection’.^11^ Consequently, symptoms associated with COVID-19 could prompt antibiotic prescriptions.

Across the world, many governments have enforced lockdown measures designed to minimise social contact and population movement, known risk factors for increased SARS-CoV-2 transmission. The UK Government imposed a nationwide lockdown from 23 March 2020, which limited people’s movements outside their homes to essential purposes only. From 1 June 2020, certain measures were eased, with national restrictions being replaced with regional measures, based on local epidemiology. Emerging research and monitoring of healthcare services suggests that attendances for a range of non-COVID-19 conditions substantially declined during the period corresponding with national lockdown. There have been reductions in diagnoses of mental health, circulatory system diseases, type 2 diabetes,^12^ major cancers,^13^ and several infectious diseases, including both acute and upper RTIs.^14^

**Table:** How this fits in

Antibiotic prescribing in primary care accounts for the majority of medical antibiotic use, significantly contributing to antimicrobial resistance. The COVID-19 pandemic changed the context for antimicrobial stewardship in primary care. Diagnoses of common conditions in primary care decreased substantially between March 2020 and May 2020. This study found that months during the pandemic period were initially associated with increased antibiotic prescribing, which then fell below expected levels during the national lockdown, highlighting that antibiotic stewardship priorities appear not to have been neglected because of COVID-19.

This research aimed to evaluate the impact of the COVID-19 pandemic on antimicrobial stewardship in primary care. The authors conducted a cohort study with interrupted time-series analysis using electronic health records to evaluate antibiotic prescribing during the pandemic. It was hypothesised that the pandemic might be associated with heightened antibiotic prescribing because of a rise in patients presenting with respiratory symptoms. Therefore, the authors assessed the proportions of RTI and UTI consultations associated with antibiotic prescribing to account for possible reductions in consultations during the pandemic period.

## METHOD

### Study population and data sources

This population-based cohort study employed UK Clinical Practice Research Datalink (CPRD) GOLD, a primary care database of anonymised electronic health records for general practices in the UK. CPRD GOLD has high coverage and good representativeness, with 11.3 million patients: an estimated 7% of the UK population.^15^ Several studies confirm the high quality of CPRD GOLD data.^16^ CPRD GOLD includes coded recording of prescriptions and clinical diagnoses from general practice, in addition to referrals to and discharge letters from secondary care.

The authors used a stratified sampling approach to randomly select registered patients from the CPRD GOLD October 2020 release, stratifying by year between 2017 and 2020, general practice, age, and sex. The start year of 2017 was selected to provide 3 years of pre-pandemic data for comparison with pandemic trends. This ensured equal representation of all general practices in the database, and that age-specific rates would be estimated with equal precision. The authors selected 12 patients from each stratum, using the ‘sample’ function in the R program (version 3.6.3), resulting in a total sample of 257 681 individual participants registered at 319 general practices contributing person-time between January 2017 and September 2020. In addition, the authors sampled and analysed separately all patients with suspected or confirmed COVID-19 from 29 January 2020 to 30 September 2020.

### Main measures

The authors calculated person-time at risk for the antibiotic prescribing sample from the start to the end of the patient’s record. Person-time was aggregated by sex and age group from 0–4 years, 5–9 years, 10–14 years, and then in 10-year age groups up to ≥85 years.

Antibiotic prescriptions were evaluated using product codes for antibiotics listed in Section 5.1 of the *British National Formulary* (BNF), with the exception of methenamine and drugs for tuberculosis and leprosy.^17^ Antibiotic prescriptions on the same date were considered as a single prescription. RTI consultations were evaluated using a list of 374 Read codes. UTI consultations were evaluated using a list of 57 Read codes.^8^^,^^9^

COVID-19 events were identified from Read codes for confirmed or suspected COVID-19 recorded in patients’ clinical, referral, and test records: ‘suspected disease caused by 2019-nCoV (novel coronavirus)’, 39%; ‘telephone consultation for suspected 2019-nCoV (novel coronavirus)’, 18%; ‘suspected coronavirus infection’, 16%; ‘2019-nCoV (novel coronavirus) detected’, 14%; ‘disease caused by 2019-nCoV (novel coronavirus)’, 5%; ‘coronavirus infection’, 4%; ‘coronavirus nucleic acid detection’, 3%; ‘[X] coronavirus infection, unspecified’, <1%; and ‘coronavirus as cause of disease classified to other chapters’, <1%. The authors excluded 278 COVID-19 diagnoses and mortality events dated on or before 29 January 2020, the official date of the UK’s first confirmed COVID-19 case. Patients with antibiotic prescriptions (from the BNF 5.1 list) during a COVID-19 episode, defined as 14 days before the index COVID-19 diagnosis date and up to 28 days following this date, were identified based on the estimated incubation period and the Public Health England definition of COVID-19 death.^18^

### Analysis

The authors calculated age- and sex-specific rates of antibiotic prescribing and rates of RTI and UTI consultations per 1000 patient–months. Rates were age-standardised using the European standard population for reference. Antibiotics prescribed to patients on the same date as their RTI/UTI consultations and estimated age-specific trends in proportions of consultations with associated antibiotic prescriptions were identified.

The authors conducted an interrupted time-series analysis to evaluate changes in antibiotic prescriptions and infection consultations.^19^ Hierarchical Poisson regression models were fitted to estimate the relative rate of antibiotic prescriptions or infection consultations for each month during the pandemic period, compared with the pre-pandemic period as reference (January 2017 to January 2020). Estimates were obtained for each month during the pandemic period from February 2020 to September 2020, adjusting for age group, sex, the secular trend over study months, and season in terms of calendar month as a factor in the model. An equivalent linear model was fitted for age-standardised rates. Coefficients from the regression models were used to predict a counterfactual scenario in which no COVID-19 pandemic took place. The fitted estimates for the pandemic and counterfactual scenario were plotted for males and females, enabling visualisation of changes.

Requests for access to data from the study should be addressed to the corresponding author at emma.rezel-potts@kcl.ac.uk. All proposals requesting data access will require approval from CPRD before data release.

## RESULTS

Data were analysed for the period January 2017 to September 2020 (Table 1). There were 253 655 registered patients in 2017 declining to 232 218 in 2020. The authors analysed data for 559 461 antibiotic prescriptions, 216 110 RTI consultations, and 36 402 UTI consultations. A total of 25 889 patients were identified with COVID-19 events from 29 January 2020 to 23 September 2020. Among these, 2942 (11%) had an antibiotic prescription dated within a COVID-19 episode (from 14 days before the index date to 28 days post-index date).

**Table 1.** Numbers of patients, antibiotic prescriptions, respiratory tract infection consultations, and urinary tract infection consultations, by year

	**2017**	**2018**	**2019**	**2020^a^**
**Total patients, *n***	253 655	251 540	244 720	232 218
**Antibiotic prescriptions, *n* (%)**	165 092 (65)	157 879 (63)	149 207 (61)	87 283 (38)
**RTI consultations, *n* (%)**	76 206 (30)	63 282 (25)	58 137 (24)	18 485 (8)
**UTI consultations, n (%)**	10 291 (4)	10 512 (4)	10 406 (4)	5193 (2)

a Record complete until September for the year 2020. RTI = respiratory tract infection. UTI = urinary tract infection.

**Figure 1.**
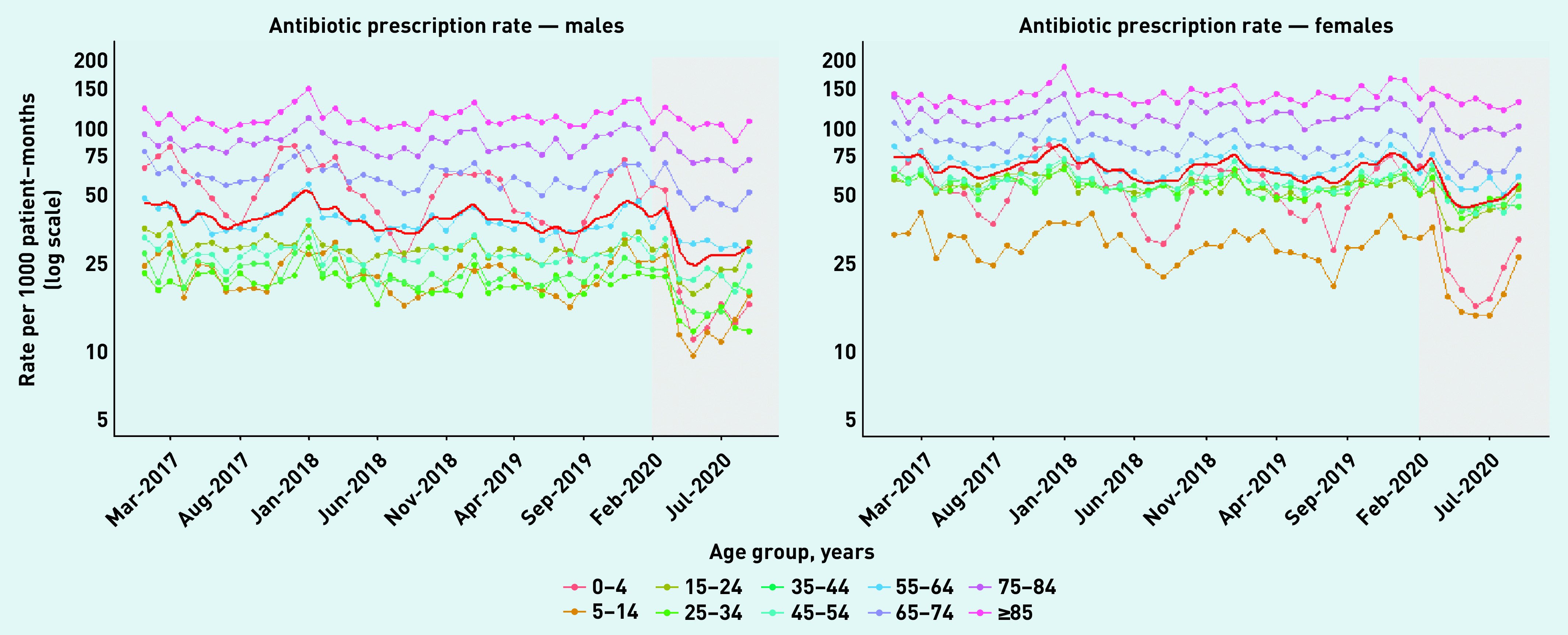
***Age- and sex-specific total antibiotic prescribing rates for males (left) and females (right) with fitted LOESS curves (solid red line), January 2017 to pandemic period: February to September 2020 (grey).***

Figure 1 shows age- and sex-specific rates of all antibiotic prescriptions by month from January 2017 to September 2020. The fitted LOESS curve (red line) represents the mean across age groups. As expected, antibiotic prescribing rates were higher in females, older adults (≥65 years), and very young children (0–4 years), but were lower in males, young adults (15–24 years), and older children (5–14 years). During the pandemic period, shaded in grey, there was a marked decline in overall antibiotic prescriptions, which was more pronounced in males, and in children (0–14 years) and young adults (15–24 years). The decline in antibiotic prescriptions during the pandemic period was less pronounced in older adults (≥65 years). Annual age- and sex-standardised rates of antibiotic prescribing are shown in Supplementary Figure S1.

**Figure 2.**
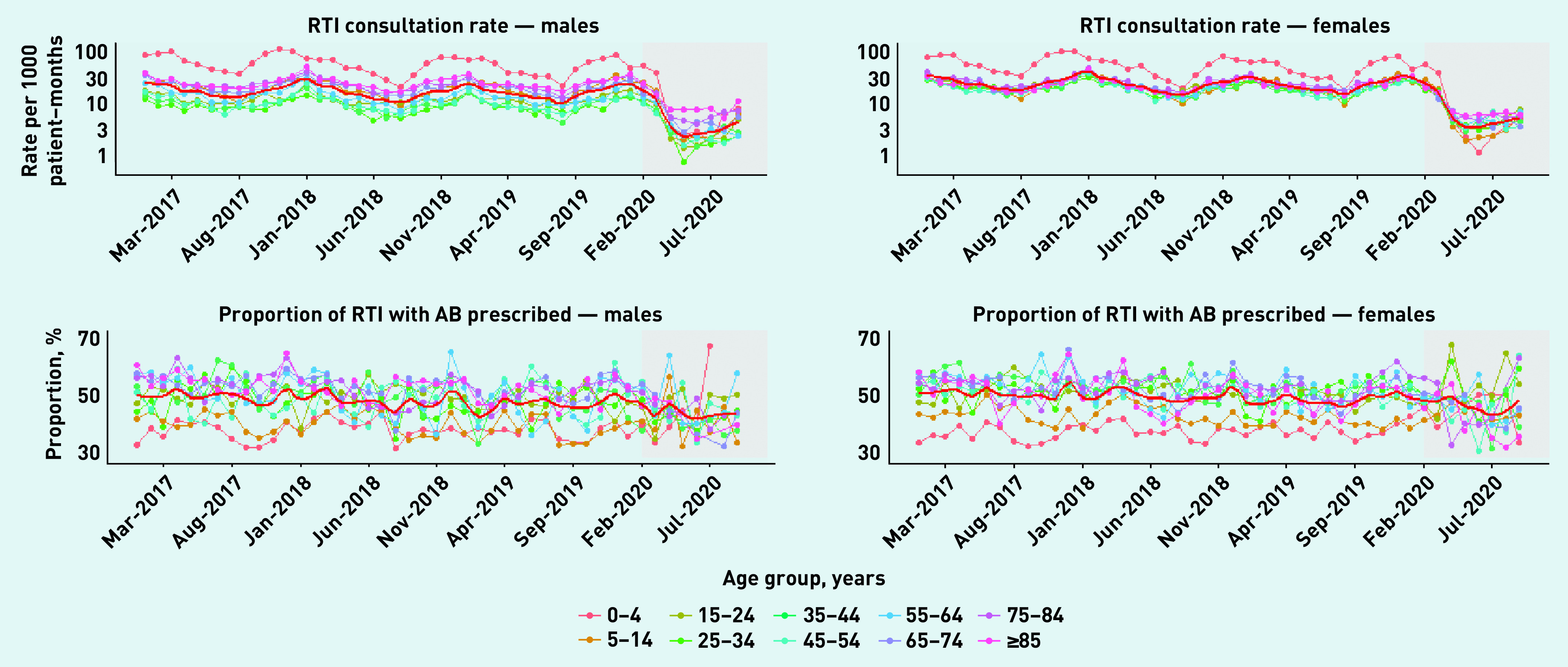
***Age- and sex-specific rates per 1000 patient–months of consultations for respiratory tract infections (top) and proportions of consultations with associated antibiotic prescriptions (bottom) for males (left) and females (right) with fitted LOESS curves (solid red line), January 2017 to pandemic period: February to September 2020 (grey).*** ***AB = antibiotic. RTI = respiratory tract infection.***

Figure 2 (upper panels) shows rates of RTI consultations per 1000 patient–months (Supplementary Figure S2 shows equivalent data for UTI consultations). There was a marked decline in RTI consultation rates during the pandemic period, which affected both sexes and all age groups. The lowest rate across the study period was in May 2020 among males aged 25–34 years, at 0.70 per 1000 patient–months. The lower panels in Figure 2 show the proportions of RTI consultations with antibiotics prescribed. The LOESS curves in these plots indicate a more limited decline in the proportion of RTI consultations with antibiotics prescribed during the pandemic period. The proportion of RTI consultations with antibiotics prescribed for females aged 45–54 years decreased to 30% in June 2020, but then increased to 63% by September 2020, compared with 54% in the same month of the previous year. UTI consultation rates and proportions of UTI consultations with antibiotics prescribed show comparatively less marked changes during the pandemic period (see Supplementary Figure S2).

**Figure 3.**
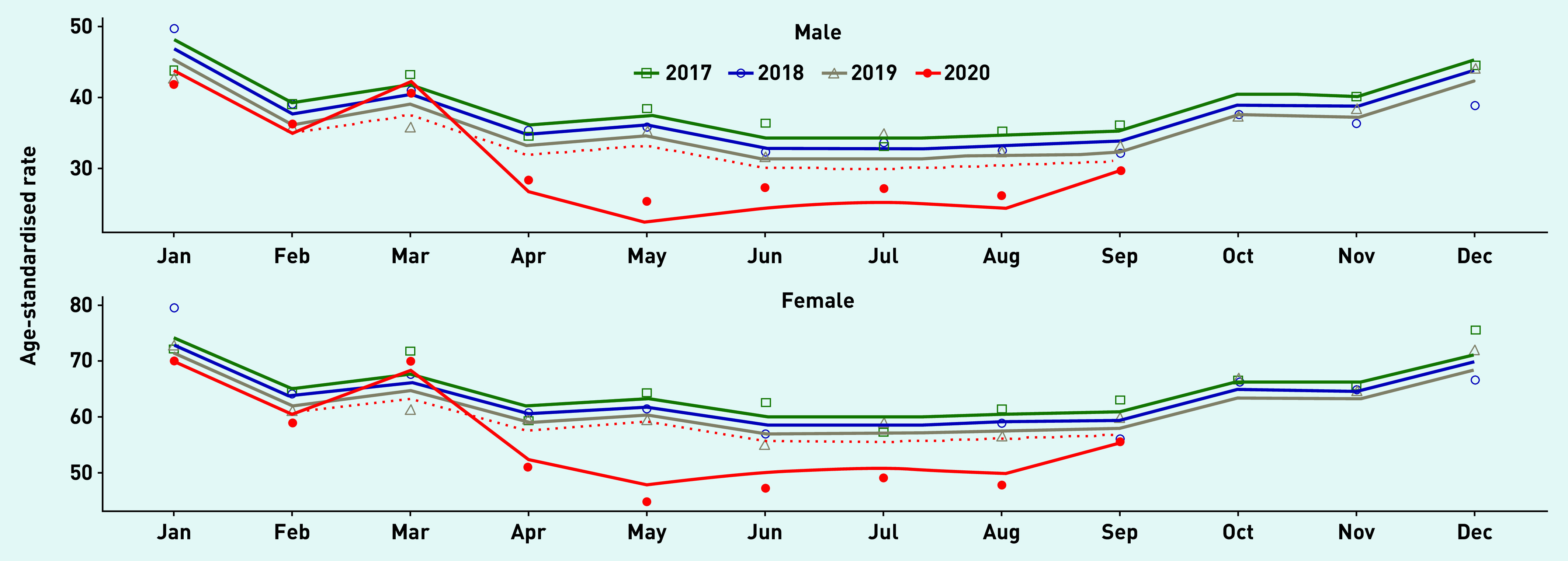
***Interrupted time-series analysis for sex- and age-standardised antibiotic prescribing, adjusted for seasonal and secular trends, January 2017 to September 2020, showing COVID-19 pandemic onset from February 2020 and counterfactual scenario (dotted red line).***

Figure 3 shows age- and sex-standardised antibiotic prescribing rates for each year of the study period. There were year-on-year declines in antibiotic prescribing. During 2020, there was a transient increase in antibiotic prescribing following the onset of the COVID-19 pandemic, which saw March 2020 rates exceeding those observed in the same months in all previous years of study. However, from April 2020 the rates were substantially lower than those predicted in the counterfactual scenario. Rates appeared to approach expected levels by September 2020.

Table 2 shows rate ratios for each month during the pandemic period compared with the pre-pandemic period for reference, adjusted for age, sex, secular trend, and season. The second month during the pandemic period, March 2020, was associated with a higher total antibiotic prescribing (adjusted rate ratio [ARR] 1.13; 95% confidence interval [CI] = 1.11 to 1.16). All other months during the pandemic period were associated with lower rates of antibiotic prescribing, particularly May 2020 (ARR 0.73; 95% CI = 0.71 to 0.75). All months during the pandemic period were associated with lower rates of RTI and UTI consultations, with the lowest rate in April for RTI consultations (ARR 0.23; 95% CI = 0.22 to 0.25) and May for UTI consultations (ARR 0.59; 95% CI = 0.55 to 0.63). The months during the pandemic period were generally associated with slightly lower proportions of RTI consultations with antibiotics prescribed, but the upper limits for most estimates were close to the null hypothesis. The lowest rate was in July (ARR 0.82; 95% CI = 0.74 to 0.91), and no associations were detected in February, April, or September. Similarly, most months during the pandemic period were not associated with a change in the proportion of UTI consultations with antibiotics prescribed.

**Table 2.** Interrupted time-series analysis showing adjusted relative rates for each month during the pandemic period compared with pre-pandemic period as reference

**Month during the pandemic period**	**Total AB prescribing (95% CI)^a^**	**RTI consultations (95% CI)^a^**	**Proportion of RTI consultations with AB prescribed (95% CI)^b^**	**UTI consultations (95% CI)^a^**	**Proportion of UTI consultations with AB prescribed (95% CI)^b^**
Pre-pandemic	Ref	Ref	Ref	Ref	Ref
Feb 2020	0.89 (0.87 to 0.91)	0.84 (0.82 to 0.87)	0.99 (0.95 to 1.03)	0.87 (0.83 to 0.92)	1.01 (0.96 to 1.07)
March 2020	1.13 (1.11 to 1.16)	0.81 (0.78 to 0.84)	0.93 (0.89 to 0.98)	0.77 (0.72 to 0.81)	1.05 (0.99 to 1.11)
April 2020	0.82 (0.80 to 0.84)	0.23 (0.22 to 0.25)	1.01 (0.93 to 1.09)	0.61 (0.58 to 0.65)	1.09 (1.02 to 1.16)
May 2020	0.73 (0.71 to 0.75)	0.16 (0.15 to 0.18)	0.86 (0.78 to 0.95)	0.59 (0.55 to 0.63)	1.05 (0.98 to 1.12)
June 2020	0.87 (0.85 to 0.89)	0.26 (0.24 to 0.28)	0.88 (0.80 to 0.97)	0.66 (0.62 to 0.70)	1.04 (0.98 to 1.11)
July 2020	0.88 (0.86 to 0.91)	0.28 (0.26 to 0.30)	0.82 (0.74 to 0.91)	0.68 (0.64 to 0.73)	1.01 (0.95 to 1.08)
Aug 2020	0.86 (0.84 to 0.89)	0.32 (0.29 to 0.34)	0.90 (0.82 to 0.98)	0.63 (0.59 to 0.67)	1.04 (0.97 to 1.11)
Sep 2020	0.91 (0.88 to 0.95)	0.30 (0.27 to 0.33)	0.98 (0.87 to 1.11)	0.72 (0.65 to 0.78)	1.05 (0.96 to 1.16)

a Adjusted for age group, sex, seasonal, and secular trends, with general practice as a random-effects variable and log of person-time as offset.

b Adjusted for age group, sex, seasonal, and secular trends, with general practice as a random-effects variable and log of consultation count as offset. AB = antibiotic. CI = confidence interval. RTI = respiratory tract infection. UTI = urinary tract infection.

## DISCUSSION

### Summary

This large population-based study explored trends in general practice antibiotic prescribing from 2017 up to, and including, the first 8 months of the COVID-19 pandemic period (February 2020 to September 2020). The first wave of the pandemic was associated with increased total antibiotic prescribing. During the period of nationwide lockdown, total antibiotic prescribing rates were considerably below rates predicted for a counterfactual scenario in which the pandemic had not taken place. RTI/UTI consultation rates declined substantially across the pandemic period, particularly among younger age groups. When patients attended for RTI consultations there was evidence of slightly reduced antibiotic prescribing, but this was not evident in three of the eight months during the pandemic. When attending for UTI consultations there was no evidence that the likelihood of antibiotic prescription was reduced across most of the months during the pandemic. This is consistent with the downturn in infection consultations being the main driver of reduced antibiotic prescribing. The authors found that 11% of patients with a suspected or confirmed COVID-19 episode received an antibiotic prescription. Comparing this with evidence that the proportion of primary care RTI consultations resulting in antibiotic prescriptions is usually about 50%^20^ gives some reassurance that indiscriminate antibiotic prescribing for COVID-19 patients did not generally feature in general practice settings.

### Comparison with existing literature

Population-based research quantifying indirect effects of consultations and prescribing in primary care during the COVID-19 pandemic have been limited. To the authors’ knowledge, this is the first UK-wide population-based study to assess and quantify its effect on antibiotic prescribing. Williams *et al* conducted a retrospective cohort study using routinely collected primary care data from the Salford Integrated Record from January 2010 to May 2020, estimating a 50% (95% CI = 41% to 57%) reduction in common mental health diagnoses compared with expected numbers between March 2020 and May 2020.^12^ Also estimated were a 43% (95% CI = 30% to 54%) reduction in diagnoses of circulatory system diseases, and a 49% (95% CI = 24% to 63%) reduction in type 2 diabetes diagnoses. Data from the Royal College of General Practitioners surveillance system also support the current findings that GP attendances were substantially reduced during the pandemic period, with weekly incidence of acute RTIs and upper RTIs declining during the same period as lockdown restrictions.^14^ Data from NHS Digital suggest that GP attendances fell markedly in March 2020, and were only partially replaced with remote consultations.^21^ Estimates of 7-day rolling averages of appointments in general practice indicate that at the end of May 2020 there were 524 333, compared with 671 313 at the end of May 2019.^22^ It is possible that patients with symptoms of fever or cough sought care within specially created COVID-19 hubs, contacted NHS telephone triage (NHS 111), obtained advice through the NHS 111 online coronavirus service, or attended the emergency departments in secondary care. It is also possible that those experiencing mild RTI/UTI symptoms opted to avoid healthcare attendance because of lockdown restrictions and risks of COVID-19 infection. Going forward, it will be important to establish whether the effects of reduced RTI and UTI primary care consultations include increased incidence in serious bacterial infections.

### Strengths and limitations

This study uses CPRD GOLD, a large database broadly representative of the national population in terms of age and sex, facilitating the precise detection of even small effect sizes and overall findings that are generalisable to the UK population. In contrast with aggregated prescribing data, CPRD GOLD offers data for infection consultations and antibiotic prescribing to individual patients, enabling estimation of consultation rates and the proportion of consultations with antibiotics prescribed.^6^

Interrupted time-series analysis has enabled the authors to harness this longitudinal data for quasi-experimental evaluation of the effects of an event that would not be possible to test using a randomisation approach. Nonetheless, there can be no confirmation of causality in the relationship between the COVID-19 pandemic and antibiotic prescribing from the current findings. It is possible that factors unrelated to the pandemic could have occurred during this time to influence the changes in prescribing observed. The pandemic itself is an unprecedented event that has triggered changes in government policies, clinical guidelines, and individual and social behaviour, in addition to the direct consequences of high rates of ill health and mortality. It is not possible to ascertain from this study which of these pandemic-induced shifts may have been most influential on rates of antibiotic prescribing and RTI/UTI consultations. There have been notable indirect effects on primary care, such as the temporary reduction of the current Quality and Outcomes Framework (QOF) requirements from 1 April 2020 to enable practices to prioritise workload aimed at preparing for and managing the COVID-19 outbreak.^23^ This, and the increased number of clinicians switching to remote working,^24^ could feasibly have changed recording behaviour during the pandemic. The authors employed a random-effects model that allowed for clustering by general practice. The estimated confidence intervals might be slightly too narrow if there is overdispersion of the data; however, an overdispersion model did not lead to convergence.

### Implications for research and practice

The initial months during the pandemic were associated with high levels of total antibiotic prescribing, which rapidly fell below expected levels as national lockdown restrictions were enforced, suggesting that the decline in prescribing is indicative of reduced primary care attendances. There was some initial uncertainty in the management of patients presenting with the confirmed pneumonia in the community in the context of COVID-19.^25^ The National Institute for Health and Care Excellence (NICE) issued rapid guidance on 3 April 2020 stating that *‘as COVID-19 becomes more prevalent in the community, patients presenting with pneumonia symptoms are more likely to have a COVID-19 viral pneumonia than a community-acquired bacterial pneumonia’*, and thus antibiotic prescriptions should be offered only where bacterial infections were suspected.^25^ It is recommended that there is improved preparedness in the speed of dissemination of rapid guidance in the event of further COVID-19 peaks in transmission or future pandemic scenarios.

Though the authors’ findings are reassuring that antibiotic stewardship priorities have not been neglected because of COVID-19, further study and monitoring are required as the UK enters a period of high COVID-19 transmission coupled with seasonal increases in common infections and lockdown restrictions that allow greater social interaction than those enforced from March 2020 to June 2020. The serious consequences of unnecessary or inappropriate prescribing necessitate ongoing commitment to antimicrobial stewardship, even in the context of COVID-19.
